# Complete Blood Count-Derived Inflammation Indices and Their Association With Cognitive Functions in Patients With Type 2 Diabetes Mellitus

**DOI:** 10.7759/cureus.93226

**Published:** 2025-09-25

**Authors:** Delila Ganic, Almir Fajkić, Orhan Lepara

**Affiliations:** 1 General Medicine, Policlinic UniMed, Sarajevo School of Science and Technology, Sarajevo, BIH; 2 Department of Pathophysiology, Faculty of Medicine, University of Sarajevo, Sarajevo, BIH; 3 Department of Human Physiology, Faculty of Medicine, University of Sarajevo, Sarajevo, BIH

**Keywords:** cognitive impairment, complete blood count, inflammatory indices, systemic inflammation, type 2 diabetes mellitus

## Abstract

Introduction: The risk of cognitive impairment, including dementia and moderate cognitive impairment (MCI), is higher in patients with diabetes and prediabetes. The need for early diagnosis biomarkers has increased due to the rise in the prevalence of type 2 diabetes mellitus (T2DM) and its related cognitive problems worldwide, as well as the lack of clear biochemical indicators and efficient treatments for dementia or cognitive decline. Chronic low-grade inflammation, reflected by elevated complete blood count-derived inflammatory indices (CBCIIs), has been implicated in both metabolic dysregulation and neurodegeneration. However, their relationship with cognitive impairment in T2DM remains insufficiently explored. The objective of this study was to investigate the association between CBCIIs and cognitive function in patients with T2DM.

Methods and materials: This cross-sectional observational study included 116 patients with T2DM recruited from diabetes counseling centers in the Public Institution Health Center of Sarajevo Canton, Bosnia and Herzegovina. Based on the assessed cognitive status, patients with T2DM were divided into two groups: with cognitive impairment (n= 76) and without cognitive impairment (n=40). A validated assessment tool, the Montreal Cognitive Assessment (MoCA), a quick test designed to screen for milder forms of cognitive impairment, was used for cognitive screening. Venous blood samples were analyzed for standard complete blood count parameters, from which 11 CBCIIs were calculated: neutrophil-to-lymphocyte ratio (NLR), derived NLR (dNLR), neutrophil-to-platelet ratio (NPR), neutrophil-to-lymphocyte-to-platelet ratio (NLPR), platelet-to-lymphocyte ratio (PLR), monocyte-to-lymphocyte ratio (MLR), systemic immune-inflammation index (SII), aggregate index of systemic inflammation (AISI), systemic inflammation response index (SIRI), lymphocyte-to-monocyte ratio (LMR), and monocyte-to-neutrophil ratio (MNR)..

Results: The results of our study showed that NLR, dNLR, NPR, NLPR, PLR, MLR, SII, AISI, and SIRI were significantly higher in the group of T2DM patients with cognitive impairment compared to the group without cognitive impairment. On the other hand, LMR and MNR were significantly lower in the group of T2DM patients with cognitive impairment compared to the group without cognitive impairment (p<0.05). The MoCA score was significantly negatively correlated with NLR, dNLR, NPR, NLPR, and SII, and positively with MNR (p<0.05)

Conclusion: Elevated CBCIIs are significantly associated with cognitive impairment in patients with T2DM. These inexpensive and widely available indices may serve as adjunctive markers for early cognitive screening in this population.

## Introduction

One of the most prevalent and rapidly expanding diseases in the world is diabetes mellitus, which, in the long term, can cause many organs to malfunction and fail, especially the heart, blood vessels, kidneys, eyes, and nerves. Type 2 diabetes mellitus (T2DM), which makes up more than 90% of all cases of diabetes, is characterized by decreased insulin sensitivity and relative insulin insufficiency. Diabetes-related persistent hyperglycemia can result in microvascular and macrovascular problems that could affect the brain [1,2].

There is mounting evidence that individuals with T2DM exhibit pathological alterations in both the structure and function of their brains. The risk of cognitive impairment (CI), including dementia and moderate CI (MCI), is 1.25-1.91 times higher in patients with diabetes and prediabetes [3,4]. These structural and functional brain changes are increasingly understood to involve chronic, low-grade inflammation as a mechanistic link between metabolic dysregulation and neurodegeneration [5].

Systemic inflammation is a plausible shared pathway, but most human studies rely on C-reactive protein (CRP) or cytokines, which are scientifically informative but operationally impractical. This highlights a clear gap for evaluating whether routine complete blood count (CBC)-derived inflammation indices can serve as practical markers of cognitive risk in T2DM. Chronic, low-grade systemic inflammation is a recognised hallmark of T2DM and is implicated in both vascular and neurodegenerative pathways to CI [6,7].

CBC-derived inflammation indices (CBCIIs), such as the neutrophil-to-lymphocyte ratio (NLR), platelet-to-lymphocyte ratio (PLR), systemic immune-inflammation index (SII), and systemic inflammation response index (SIRI), integrate information from different leukocyte subtypes and platelets into composite measures of systemic inflammatory activity. Unlike specialised inflammatory assays, CBCIIs are inexpensive, routinely available, and require no additional sampling [8].

Previous studies in mixed and non-diabetic cohorts have linked elevated CBCIIs with lower Montreal Cognitive Assessment (MoCA) scores and increased risk of MCI or dementia [9]. However, their performance as markers of cognitive status has not been systematically evaluated within a T2DM population.

This study aimed to examine the association between multiple CBCIIs and cognitive function in patients with T2DM, comparing those with and without CI.

## Materials and methods

This was a cross-sectional observational study conducted at the diabetes counseling centers in the Public Institution Health Center of Sarajevo Canton, Bosnia and Herzegovina. The study was conducted in accordance with the Declaration of Helsinki (Revision 2013). This study is part of a larger study (doctoral dissertation entitled “Association between Cognitive Status, C-reactive Protein, and Anthropometric Parameters in Patients with Diabetes Mellitus") that was approved by the Ethics Committee of the Public Institution Health Center of Sarajevo Canton (approval number: 01-11-33-2-2683-2/23), and informed consent was obtained from all participants.

Study population

Inclusion and Exclusion Criteria

The inclusion and exclusion criteria are summarized in Table 1.

**Table 1:** Inclusion and exclusion criteria T2DM: type 2 diabetes mellitus

Category	Criteria
Inclusion	Diagnosis of T2DM
	Age ≥ 18 years
	Both male and female patients presenting to Diabetes Counseling Centers of the Public Institution Health Center of Sarajevo Canton
Exclusion	Presence of acute or chronic inflammatory conditions
	Presence of diabetic complication (e.g. diabetic nephropathy,etc)
	Presence of neurodegenerative diseases
	Presence of asthma
	Presence of cirrhosis of the liver
	Presence of Crohn’s disease or ulcerative colitis
	Presence of malignant neoplasms
	Presence of pituitary tumors

The study included all eligible adults with a diagnosis of T2DM consecutively assessed during the study period who fulfilled the eligibility criteria and provided informed consent (n=116). Based on cognitive status, patients were divided into two groups: (a) T2DM with CI (n = 76), and (b) T2DM without CI (n = 40).

Sample size rationale

No formal a priori sample size calculation was performed. A post hoc sensitivity check, calculated using G*Power 3.1 [10], indicated that, at α=0.05 and 80% power, this sample was sufficient to detect (i) a correlation of at least ρ≈0.30 between CBCIIs and MoCA within the T2DM cohort, and (ii) a between-group difference of ~d=0.55 (moderate effect) for CBCIIs when comparing patients with vs. without CI. These thresholds align with effects reported in prior observational literature and justify the analytical focus on moderate or larger associations.

Cognitive function assessment

A validated assessment tool, the MoCA, a quick test designed to screen for milder forms of CI, was used for cognitive screening. The 30-point exam consists of a single page and evaluates skills such as working memory, short-term memory, executive function, attention, concentration, language, orientation, and visuospatial function. Normal cognitive function was defined as a score of at least 26 out of 30 [11].

Laboratory analysis

Participants' blood samples were gathered from the labs. Venous blood samples (5 mL) were collected between 7:00 and 8:00 AM following an overnight fast. Samples were processed within 24 hours and analyzed for complete blood count parameters using standard laboratory protocols on an automated hematology analyzer. Based on the laboratory test results, 11 CBCIIs were calculated, similar to a prior study [12]. They are listed in Table 2.

**Table 2:** Complete blood count-derived inflammation indices

CBCII	Parameter	Formula
NLR	Neutrophil to Lymphocyte Ratio	(neutrophils) / (lymphocytes)
dNLR	Derived Neutrophil to Lymphocyte Ratio	(neutrophils) /(white cells - neutrophils)
NPR	Neutrophil to Platelet Ratio	(neutrophils) / (platelets)
NLPR	Neutrophil to Lymphocyte and Platelet Ratio	(neutrophils / (lymphocytes × platelets)
PLR	Platelet to Lymphocyte Ratio	(platelets) / (lymphocytes)
MNR	Monocyte to Neutrophil Ratio	(monocytes) / (neutrophils)
MLR	Monocyte to Lymphocyte Ratio	(monocytes) / (lymphocytes)
LMR	Lymphocyte to Monocyte Ratio	(lymphocytes) / (monocytes)
SII	Systemic Immune Inflammation Index	(neutrophils × platelets) / (lymphocytes)
AISI	Aggregate Index of Systemic Inflammation	(neutrophils × monocytes × platelets) / (lymphocytes)
SIRI	Systemic Inflammation Response Index	(neutrophils × monocytes) / (lymphocytes)

Statistical analysis

SPSS Statistics for Windows, version 16.0 (SPSS Inc., Chicago, Illinois, United States) was used to conduct the statistical analysis. Kolmogorov-Smirnov and Shapiro-Wilk tests were used to determine whether continuous variables had a normal distribution. The Student t-test was used to assess differences between the compared groups for numerical variables that were normally distributed. Numerical variables that were not normally distributed were displayed as median and interquartile range, and the Mann-Whitney U test was used to evaluate group differences. The chi-square test was used to assess variations in the frequency of categorical variables between groups. Categorical variables were displayed as absolute numbers and as percentages. Spearman's rank correlation analysis was used to test for relationships between continuous variables. Statistical significance was defined as a p-value of < 0.05.

## Results

Table 3 summarises the baseline characteristics of patients with T2DM stratified by cognitive status. Patients with CI had a significantly longer duration of diabetes (median 15.0 vs. 10.5 years, p=0.045) and lower MoCA scores (median 22.0 vs. 27.0, p<0.001) compared with those without impairment.

**Table 3:** Baseline characteristics of patients with T2DM stratified by cognitive status Data are presented as median (interquartile range) unless otherwise indicated. *p < 0.05; **p < 0.001 SBP: systolic blood pressure; DBP: diastolic blood pressure; HbA1c: glycated hemoglobin; MoCA: Montreal Cognitive Assessment; CI: cognitive impairment; T2DM: type 2 diabetes mellitus

Variable	T2DM with cognitive impairment (n = 76)	T2DM without cognitive impairment (n = 40)	p-value
Demographics			
Gender (male/female), n (%)	22 (55.0%)/ 5 (59.2%)	18 (45.0%) / 31 (40.8%)	χ²=.0.190; df=1; p=0.663; Cramer’s V =0.041
Age, years	66.0 (59.0–72.0)	64.0 (55.25–72.75)	0.181
Education, n (%)			
Primary school	12 (15.8%)	2 (5.0%)	–
Secondary school	50 (65.8%)	20 (50.0%)	
Higher vocational education	4 (5.3%)	3 (7.5%)	
Higher education	–	15 (37.5%)	
Clinical characteristics			
Diabetes duration, years	15.0 (10.0–20.0)	10.5 (7.25–24.0)	0.045*
Smoking, n (%)	14 (35.0%)	30 (39.5%)	0.637
Drinking, n (%)	17 (42.5%)	33 (43.4%)	0.924
SBP, mmHg	140.0 (120.0–140.0)	130.0 (120.0–140.0)	0.478
DBP, mmHg	80.0 (70.0–80.0)	80.0 (72.50–80.0)	0.706
Laboratory measures			
Glucose level, mmol/L	8.17 (6.72–9.98)	7.38 (5.99–10.05)	0.217
HbA1c, %	7.90 (6.90–8.70)	7.50 (6.80–8.75)	0.270
Cognitive measure			
MoCA score	22.0 (21.0–23.0)	27.0 (26.0–28.0)	0.000**

Regarding blood cell counts, patients with CI had higher neutrophil counts (p=0.001) and lower lymphocyte counts (p=0.004). Monocyte and platelet counts did not differ significantly between groups (p=0.076 and p=0.591, respectively). All evaluated CBCIIs, except MNR and LMR, were significantly elevated in the T2DM with CI group (all p<0.05). MNR and LMR were significantly lower in this group (p=0.034 and p<0.001, respectively). These differences are summarised in Table 4.

**Table 4:** Comparison of CBC and CBCIIs between the two groups *p < 0.05; **p < 0.001 CBC: complete blood count; CBCII: complete blood count-derived inflammatory indices; NLR: neutrophil-to-lymphocyte ratio; dNLR: derived NLR; NPR – neutrophil-to-platelet ratio; NLPR: neutrophil-to-lymphocyte and platelet ratio; PLR: platelet-to-lymphocyte ratio; MLR: monocyte-to-lymphocyte ratio; MNR: monocyte-to-neutrophil ratio; LMR: lymphocyte-to-monocyte ratio; SII: systemic immune-inflammation index; AISI: aggregate index of systemic inflammation; SIRI: systemic inflammation response index; CI: cognitive impairment.

Variables	T2DM with cognitive impairment (n = 76)	T2DM without cognitive impairment (n = 40)	p-value
Neutrophils, median (IQR)	4.89 (3.87-6.01)	3.13 (2.57-4.37)	0.001*
Lymphocytes, median (IQR)	1.98 (1.70-2.48)	2.46 (2.22-2.84)	0.004*
Monocytes, mean ± SD	0.55±0.17	0.47±015	0.076
Platelets, median (IQR)	237.0 (205.25-303.25)	261.5 (210.25-292.75)	0.591
NLR, median (IQR)	2.33 (2.03-3.06)	1.30 (1.06-1.83)	0.000**
dNLR, median (IQR)	1.76 (1.53-2.28)	1.02 (0.82-1.38)	0.000**
NPR, median (IQR)	0.019 (0.014-0.024)	0.011 (0.010-0.017)	0.000**
NLPR, mean ± SD	0.015±0.004	0.010±0.002	0.000**
PLR, mean ± SD	134.70±39.89	105.63±31.90	0.004*
MLR, mean ± SD	0.28±0.09	0.18±0.006	0.003*
MNR, mean ± SD	0.11±0.03	0.13±0.004	0.034*
LMR, median (IQR)	3.51 (2.77-4.98)	5.20 (4.34-7.87)	0.000**
SII, median (IQR)	667.05 (459.50-799.20)	339.78 (267.05-429.47)	0.000**
AISI	214.76 (489.81-489.81)	155.63 (99.36-228.47)	0.000**
SIRI, median (IQR)	1.31 (0.91-1.85)	0.89 (0.61-1.40)	0.000**

In correlation analysis, the MoCA score in patients with cognitive impairment was negatively associated with neutrophil count, NLR, dNLR, NPR, NLPR, and SII, and positively associated with MNR (all p<0.05). No significant correlations were observed in the group without CI (Table 5).

**Table 5:** Correlation of MoCA score with CBC and CBCIIs in the two groups Rho = Spearman’s rank correlation coefficient. *p < 0.05; **p < 0.001 CBC: complete blood count; CBCII: complete blood count-derived inflammatory indices; MoCA: Montreal Cognitive Assessment; T2DM: type 2 diabetes mellitus; CI: cognitive impairment; NLR: neutrophil-to-lymphocyte ratio; dNLR: derived NLR; NPR: neutrophil-to-platelet ratio; NLPR: neutrophil-to-lymphocyte and platelet ratio; PLR: platelet-to-lymphocyte ratio; MLR: monocyte-to-lymphocyte ratio; MNR: monocyte-to-neutrophil ratio; LMR: lymphocyte-to-monocyte ratio; SII: systemic immune-inflammation index; AISI: aggregate index of systemic inflammation; SIRI: systemic inflammation response index

Variable	MoCA score – T2DM with cognitive impairment (n = 76)	MoCA score – T2DM without cognitive impairment (n = 40)
Neutrophils	Rho= -0.357*	Rho= -0.147
Lymphocytes	Rho= 0.300	Rho= -0.234
Monocytes	Rho= 0.112	Rho= -0.024
NLR	Rho= -0.642**	Rho= 0.184
dNLR	Rho= -0.590**	Rho= 0.200
NPR	Rho= -0.444**	Rho= 0.041
NLPR	Rho= -0.465**	Rho= 0.344
PLR	Rho= -0.156	Rho= 0.105
MLR	Rho= -0.124	Rho= 0.155
MNR	Rho= 0.421*	Rho= -0.063
LMR	Rho= 0.124	Rho= -0.155
SII	Rho= -0.407*	Rho= 0.097
AISI	Rho= -0.188	Rho= -0.074
SIRI	Rho= -0.302	Rho= 0.037

## Discussion

T2DM is a well-recognized risk factor for cognitive dysfunction, with epidemiological studies consistently showing increased prevalence of impairments in processing speed, attention, and executive function among affected individuals [13,14]. Notably, some studies suggest that even prediabetes, a state of impaired glucose regulation that precedes overt diabetes, can accelerate cognitive decline, particularly in memory and executive domains, highlighting the potential value of early metabolic screening and intervention [15].

The length of time a person has lived with diabetes appears to be a key determinant of risk. Large population datasets, including the United Kingdom Biobank [16], show that the probability of developing dementia increases steadily with disease duration, more than doubling after 15 years of diabetes compared with less than five years, even when vascular comorbidities and glycemic control are taken into account. Comparable results have been reported in the National Health and Nutrition Examination Survey (NHANES) cohort, where individuals with over two decades of diabetes had more than a threefold higher likelihood of experiencing slowed processing speed and reduced attentional capacity, independent of demographic and vascular factors [14]. Consistent findings from longitudinal research further indicate that prolonged exposure to hyperglycemia and related metabolic disturbances accelerates decline in executive abilities and information-processing efficiency [17]. Our results are in line with these observations, showing that patients with T2DM and CI had significantly longer diabetes duration than their cognitively intact counterparts.

Beyond epidemiological associations, several interrelated metabolic and inflammatory mechanisms have been proposed in previous studies to be involved in the pathogenesis of cognitive decline in T2DM. Glucose serves as the primary energy substrate for the brain, and alterations of carbohydrate metabolism (such as insulin resistance, abnormal neuronal glucose uptake, recurrent hypoglycemia, and cerebrovascular hypoperfusion) may disrupt neuronal integrity and synaptic function [18].

A growing body of studies supports the concept of “type 3 diabetes,” or brain insulin resistance, which has been suggested to contribute to impaired neuronal glucose utilization, decreased synaptic plasticity, increased tau phosphorylation, and eventually pathology like that seen in Alzheimer’s disease [19]. The consequent metabolic dysfunction may further enhance oxidative stress, lipid peroxidation, mitochondrial damage, DNA injury, and neuronal vulnerability [20]. Disruption of the blood-brain barrier (BBB) has also been implicated as a mechanism allowing peripheral inflammatory mediators and immune cells to access the central nervous system (CNS) and amplify injury. Microglial activation is thought to play a central role in this process, shifting toward a pro-inflammatory phenotype, releasing cytokines and reactive oxygen species, and perpetuating a cycle of neuroinflammation and neurodegeneration [21]. Additionally, systemic inflammation has been recognized as a significant contributing factor to metabolic dysregulation and neurodegeneration in the pathogenesis of T2DM. Macrovascular and microvascular complications, in addition to CI in the elderly, have been linked to changes in hematological indices, particularly increased total leukocyte count [22,23]. Our study did not directly evaluate these mechanisms; they are presented here solely for biological plausibility based on prior literature.

Our results demonstrated that several CBCIIs, namely NLR, dNLR, NPR, NLPR, PLR, MLR, SII, AISI, and SIRI, were significantly higher in T2DM patients with CI, whereas LMR and MNR were lower. Furthermore, MoCA scores correlated negatively with NLR, dNLR, NPR, NLPR, and SII, and positively with MNR. These findings align with prior studies that have linked NLR and related indices to increased risk of CI in T2DM patients [24]. Given the cross-sectional nature of both our study and most prior research, these associations should not be interpreted as causal relationships but rather as evidence of a potential inflammatory signature accompanying CI in T2DM.

Previous studies have suggested that neutrophil-driven inflammation may contribute to neuronal injury through oxidative stress, release of pro-inflammatory cytokines, and modulation of amyloid and tau pathology, while lower lymphocyte counts may indicate compromised adaptive immunity [25].

One hypothesis-generating mechanism described in the literature involves the formation of Neutrophil Extracellular Traps (NETs)-web-like chromatin structures enriched in proteases and myeloperoxidase, which, besides playing an antimicrobial role, may induce collateral damage to the surrounding neurons and glial cells. NETs are considered a strong source of reactive oxygen species (ROS), potentially amplifying oxidative stress and promoting endothelial dysfunction in cerebral microvessels [26].

Meanwhile, lymphocytes, particularly the regulatory T cells (Tregs), are thought to exert neuroprotective effects through the attenuation of microglial activation and resultant neuroinflammation. A reduced number or dysfunctional Tregs in T2DM has been proposed to facilitate chronic activation of pro-inflammatory pathways and hence neuronal loss [27]. Elevated PLR, NLR, and SII have been associated with hippocampal amyloid deposition and brain atrophy [28]. In this context, markers such as SIRI and SII might serve as accessible, cost-effective biomarkers for identifying patients at increased risk of cognitive decline.

Our study did not directly measure these mechanisms; they are discussed here solely for biological plausibility and as hypotheses to be explored in future longitudinal research.

From a clinical perspective, our findings support the potential integration of CBCIIs into routine screening protocols for patients with T2DM who are at increased risk of cognitive decline, particularly those with longer diabetes duration (>10-15 years), suboptimal glycemic control, coexisting vascular risk factors (e.g., hypertension, dyslipidemia), or prior cardiovascular/cerebrovascular events [29]. These markers are inexpensive, widely available through standard complete blood counts, and easily repeatable over time, making them practical for longitudinal monitoring in primary care and endocrinology settings. Optimal risk stratification would combine CBCIIs with validated cognitive screening tools (e.g., MoCA) and, where feasible, adjunctive assessments such as structural or functional neuroimaging. This multimodal approach could potentially enable earlier detection of subtle cognitive changes, timely intervention, and more individualized management strategies in T2DM populations.

Longitudinal studies are warranted to observe changes in CBCIIs over time, from the period of mild CI to overt dementia in T2DM patients. Such studies will eventually answer the question of whether these indices start changing before the clinical manifestation of decline and thus offer a period for intervention. Plasma or CSF neurofilament light chain, amyloid/tau PET imaging, and MRI-based volumetric assessments are some of the emerging biomarkers of neurodegeneration with which hematological indices may be integrated to fine-tune risk stratification further. In addition to these biomarkers, digital cognitive testing platforms can evolve into a scalable, inexpensive yet multifactorial screening approach pertinent to clinical practice as well as large-scale epidemiological research.

Strengths of this study include the use of a well-validated cognitive assessment tool (MoCA), the analysis of multiple inflammation-based indices, and the recruitment of a real-world clinical population. Limitations of this study include the single-center, cross-sectional design and absence of longitudinal follow-up, which preclude causal inference. The study relied solely on cognitive screening tools (MoCA) without confirmation of CI using brain MRI, CSF biomarkers, or other neuroimaging modalities. Furthermore, there was no non-diabetic control group, limiting broader population comparisons and preventing assessment of confounding effects across different populations. Additional limitations include the lack of adjustment for education level, socioeconomic status, and potential medication effects (e.g., statins, antihypertensives, and antidiabetic agents with anti-inflammatory properties). No correction for multiple testing across several inflammatory indices was applied. Finally, recruitment from diabetes counseling centers alone may introduce selection bias and limit generalizability to the broader T2DM population.

## Conclusions

In patients with T2DM, CBCIIs were found to be significantly elevated in the presence of CI compared to diabetics with preserved cognitive function. These findings suggest a possible role of systemic inflammation in the development of CI in this population but do not establish causality due to the cross-sectional design. The observed negative correlations between several inflammatory indices and MoCA scores further highlight an association between systemic inflammation and cognitive status. Elevated inflammatory indices may reflect chronic, low-grade inflammation linked to cerebrovascular and neurodegenerative vulnerability. Monitoring these routinely available laboratory markers could potentially assist in early identification of high-risk patients; however, prospective longitudinal studies with standardized biomarker assessments, multimodal cognitive testing, and neuroimaging confirmation are warranted to clarify temporal relationships and evaluate potential therapeutic strategies aimed at modulating systemic inflammation.
